# A DNA methylation signature discriminates between excellent and non-response to lithium in patients with bipolar disorder type 1

**DOI:** 10.1038/s41598-020-69073-0

**Published:** 2020-07-22

**Authors:** C. Marie-Claire, F. X. Lejeune, E. Mundwiller, D. Ulveling, I. Moszer, F. Bellivier, B. Etain

**Affiliations:** 1 Optimisation thérapeutique en Neurospsychopharmacologie, INSERM U1144, Université de Paris, Paris, France; 2 AP-HP, GH Saint-Louis—Lariboisière—F. Widal, Pole de Psychiatrie Et de Médecine Addictologique, Paris, France; 3 Fondation FondaMental, Créteil, France; 4 IGenSeq, Institut du Cerveau Et de La Moelle Épinière, Paris, France; 5 Bioinformatics and Biostatistics Core Facility iCONICS, Inserm U 1127, CNRS UMR 7225, Sorbonne Université UMR S 1127, Institut du Cerveau Et de La Moelle Épinière, Paris, France; 6 Faculté de Médecine, Université de Paris, Paris, France

**Keywords:** DNA methylation, Diagnostic markers, Bipolar disorder

## Abstract

Lithium (Li) is the cornerstone maintenance treatment for bipolar disorders (BD), but response rates are highly variable. To date, no clinical or biological marker is available to reliably define eligibility criteria for a maintenance treatment with Li. We examined whether the prophylactic response to Li (assessed retrospectively) is associated with distinct blood DNA methylation profiles. Bisulfite-treated total blood DNA samples from individuals with BD type 1 (15 excellent-responders (LiERs) versus 11 non-responders (LiNRs)) were used for targeted enrichment of CpG rich genomic regions followed by high-resolution next-generation sequencing to identify differentially methylated regions (DMRs). After controlling for potential confounders we identified 111 DMRs that significantly differ between LiERs and LiNRs with a significant enrichment in neuronal cell components. Logistic regression and receiver operating curves identified a combination of 7 DMRs with a good discriminatory power for response to Li (Area Under the Curve 0.806). Annotated genes associated with these DMRs include Eukaryotic Translation Initiation Factor 2B Subunit Epsilon (*EIF2B5*), Von Willebrand Factor A Domain Containing 5B2 (*VWA5B2*), Ral GTPase Activating Protein Catalytic Alpha Subunit 1 (*RALGAPA1*). Although preliminary and deserving replication, these results suggest that biomarkers of response to Li may be identified through peripheral epigenetic measures.

## Introduction

An early age at onset, a high rate of mood recurrences, the associated medical health and psychosocial burdens make bipolar disorder (BD) one of the leading causes of disability in the young population^1,2^. Consensus conferences and experts’ guidelines recommend lithium (Li) as a first-line prophylactic treatment for BD^3^. Indeed, Li has proven its efficacy for treating acute manic episodes^4^, for preventing mood relapses of any polarity^5,6^, and also for preventing suicidal behaviors^5^.

Predicting response to Li in BD is crucial to move towards a more personalized medicine^7^. Indeed, not all patients receiving Li for at least two cumulative years of treatment will display improvement in the frequency and/or severity of mood recurrences. In individuals with BD who received Li, three subpopulations (full or excellent responders, partial responders and non-responders) have been repeatedly identified, with around one third of the patients belonging to each group^8–10^.

Considerable research effort has been dedicated to the identification of clinical predictors of a ‘good response’ to Li, however no definite eligibility criteria for Li treatment has been identified^11^. For example, Hui and colleagues used a meta-analytic approach to suggest six predictors of good response to Li: mania-depression-interval sequence, absence of rapid cycling, absence of psychotic symptoms, family history of bipolar disorder, shorter pre-lithium illness duration and later age of onset of BD^12^. Unfortunately, these variables exhibited small to moderate effect sizes, likewise low negative and positive predictive values, and cannot be reliably used for stratification and personalized approaches.

In this context, the identification of biological markers that are associated with the response to Li represents a mandatory first step towards a personalized medicine. Among biological markers, those related to epigenetic marks might prove to be of interest in BD^13^. Indeed, epigenetic mechanisms (such as DNA methylation and histone acetylation) represent adaptive patterns of gene expression that might result from and/or drive the effects of medications^14^. These markers might serve as a tool to fill the gap between empirical prescriptions with unpredictable response and a more personalized and effective prescription of Li.

The investigation of epigenetic marks applied to the response to Li in BD is very recent, with most of the available studies being published in the last 5 years. A few studies have focused on global DNA methylation in lymphoblastoid cell lines or leukocytes from patients with BD, suggesting a decrease in global methylation in patients who responded to Li^15,16^. A few other studies have investigated candidate genes and reported a hypomethylation at the *BDNF* (Brain-derived neurotrophic factor) promoter in peripheral blood mononuclear cells (PBMC) in patients treated with Li^17–19^. Genome-wide analysis of the influence of Li on the methylome of the neuroblastoma human cell line SK-N-SH found that Li modulated the methylation level at several CpG sites^20^. Finally, one genome-wide analysis of DNA methylation in patients with BD found that, unlike quetiapine and valproic acid, Li did not significantly influence DNA methylation after correction for blood cell type composition^21^. However, further global methylation studies are required to investigate how response to Li might be related to methylation status.

To identify a DNA methylation signature of response to Li in individuals with BD type 1, we therefore performed a genome-wide methylation study of whole blood native DNA comparing excellent responders (LiERs) versus non-responders (LiNRs) to identify differentially methylated regions (DMRs).

## Results

As shown in Table 1, LiERs (N = 15) and LiNRs (N = 11) were similar for gender, age at inclusion, BMI and smoking status. LiNRs received more frequently atypical antipsychotics as compared to LiERs (p = 5.0 10^–3^, one-sided Fisher’s exact test) and tended to receive more drugs (p = 0.051, one-sided Fisher’s exact test). As expected, as compared to LiNRs, LiERs were predominantly under current Li medication (p = 0.011, one-sided Fisher’s exact test).

**Table 1:** Clinical characteristics of the LiER and LiNR patients with bipolar disorder included in the global methylation study.

	**LiER**	**LiNR**	**P-value**
N	15	11	
Alda range	7–10	0–3	
Ratio Male/Female	8/7	6/5	0.95
Age	49.85 ± 11.24	46.58 ± 6.54	0.32
BD type 1	100%	100%	
BMI	24.48 ± 3.26	25.71 ± 3.16	0.35
Smokers yes/no	6/9	6/5	0.47
Current medication			
Li yes/no	14/1	5/6	7.6 10^–3^
Anticonvulsants yes/no	3/12	5/6	0.17
Atypical antipsychotic yes/no	0/15	7/4	3.9 10^–4^
Antidepressants yes/no	3/12	4/7	0.36
Number of psychotropic drugs 1/2/3	11/3/1	3/4/4	0.015

*Li* lithium, *ER* excellent responder, *NR* non-responder, *N* number, *BD* bipolar disorder, *BMI* body mass index.

Blood cell-type compositions were estimated from the DNA methylation profile using 479 CpGs (Supplementary Table 1). As shown in Supplementary Fig. 1, there is no significant difference in estimated cell-type percentages between LiERs and LiNRs (Welch two-sample t-test, p > 0.05).

### Differentially methylated regions in LiERs and LiNRs and enrichment analysis

**Figure 1:**
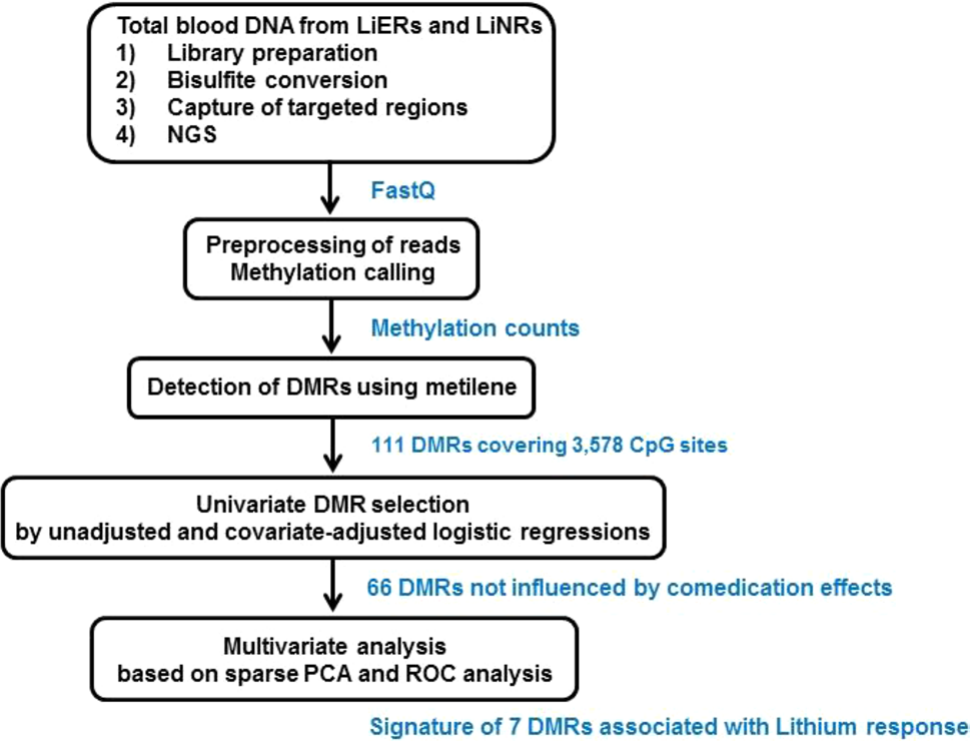
Bioinformatic analysis flowchart used in this study for the identification of a DNA methylation signature in LiER *vs* LiNR patients with bipolar disorder type 1.

**Figure 2:**
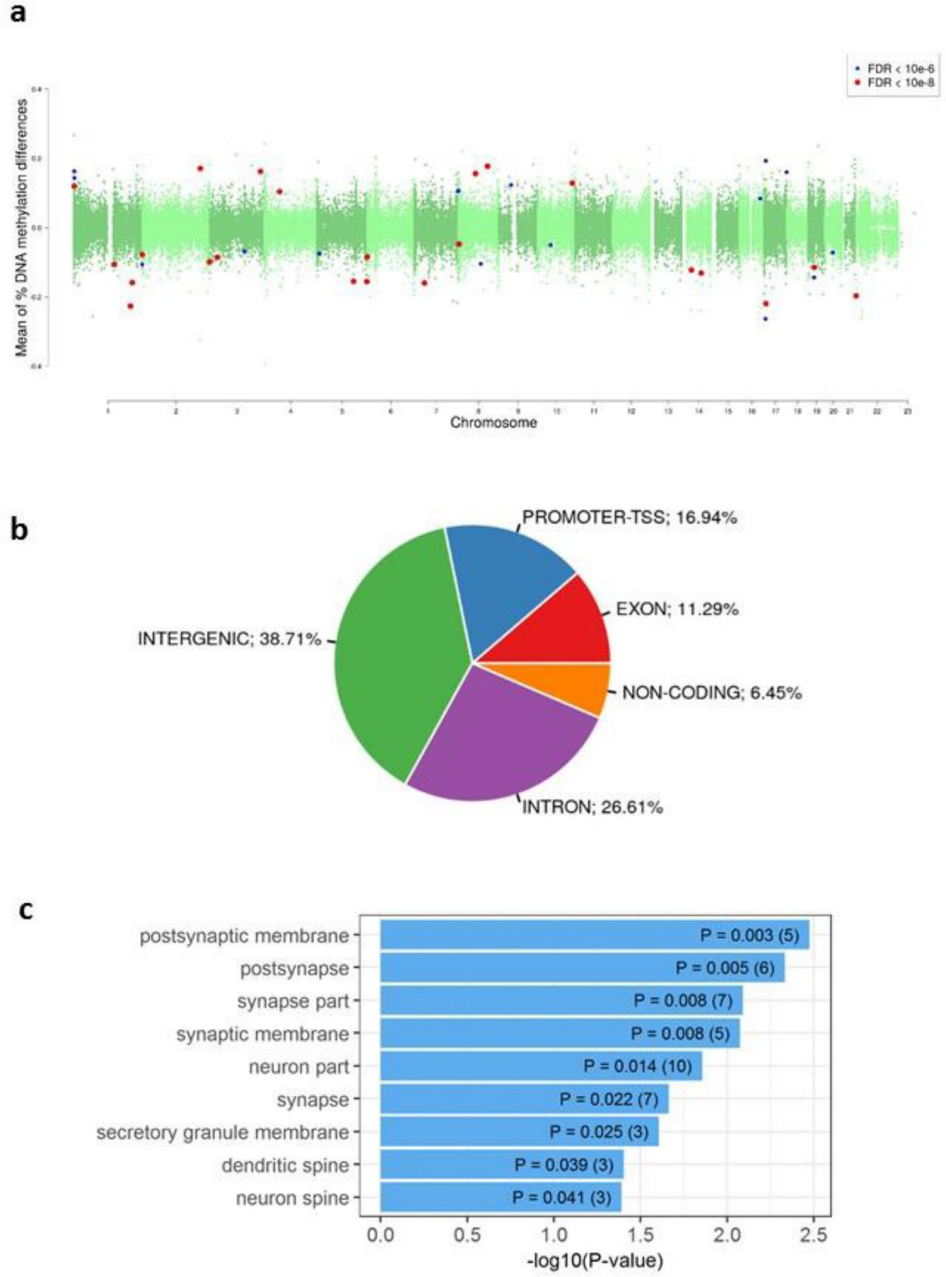
(**a**) Manhattan plots showing the distribution of p-values of DMRs associated with Li response across chromosomes. (**b**) Representation of genomic locations of significant DMRs in LiER *vs* LiNR. TSS: transcription start site (**c**) Significant GO terms (cel components) associated with the 111 significant DMRs. The p value and (number of genes) in each category are presented in the bars.

We investigated if there were differences in DNA methylation in the LiERs compared to the LiNRs. Figure 1 shows a flowchart illustrating the DMR selection process. One hundred and eleven DMRs, spanning 3,578 CpG sites, were found to be significantly associated to response to Li (FDR < 0.05). The locations of the DMRs across the genome are presented in a modified Manhattan plot (Fig. 2a) indicating the mean DNA methylation differences between LiERs and LiNRs. The DMRs with the most significant FDR (FDR < 10^–6^) are distributed on 14 chromosomes. As shown in Fig. 2b, 17% of the DMRs were located in promoter regions, 39% were intergenic, 11% were exonic and 27% were intronic.

GO cellular component and KEGG pathway enrichment analyses were performed using DAVID 6.8 for the complete list of the 74 genes associated with the 111 DMRs (Supplementary Table 2). Nine enriched GO-CC terms were identified, eight of them being related to neuronal cells. The significant terms ranked by p-values and the number of genes in each GO item are presented in Fig. 2c. One enriched KEGG pathway was also identified (Synthesis and degradation of ketone bodies, p = 0.023). However none of these enriched pathway or cell component remained significant after correction.

### Potential effects of associated medications

Since the two groups of patients differ in terms of medications (LiNRs received more frequently atypical antipsychotics, more psychotropic drugs and less frequently Li), the next step of the analytic strategy was to investigate the potential confounding effects of medications in the relationship between the response to Li and the mean methylation level of DMRs (Fig. 1). Out of the 111 DMRs, 91 were selected with p < 0.1 in univariate logistic regressions, of which 25 were excluded due to a potential confounder effect of medications as indicated by comparison of the crude and adjusted OR estimates. Therefore, 66 DMRs fairly associated with the response to Li, independently of the comedication status (atypical antipsychotics, antidepressants, anticonvulsants and number of psychotropic drugs), but also current Li treatment, and were then considered for the next step of the multivariate analysis.

### Identification of an optimal combination of DMRs to discriminate LiERs and LiNRs

**Figure 3:**
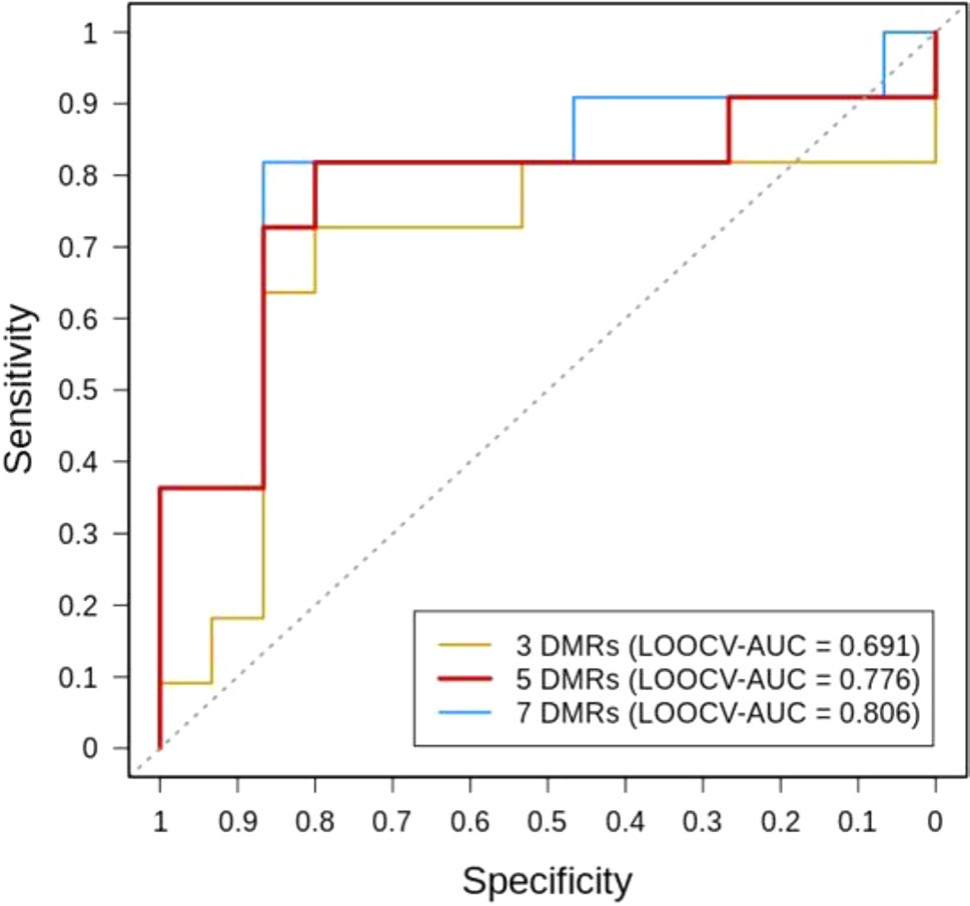
Receiver operating characteristics (ROC) of the combinations of DMRs. Area under the ROC curve for LIER *vs* LiNR of the sequential combinations of: 3 DMRs model, yellow line; 5 DMRs model, red line; 7 DMRs model, blue line.

To further investigate the potential of a DMR signature to associate to the response to Li, sPLS-DA was performed with the 66 selected DMRs using a stepwise procedure combining the Lasso selection and ROC curve analysis (Table 2). Based on the first component, a linear combination of the candidate DMRs was built by adding each DMR, one at a time, until the LOOCV-AUC criteria can be maximized with the most stable selection of DMRs possible. As shown in Fig. 3, the classification of LiERs and LiNRs with DMR67206, DMR24332 and DMR30347 (N = 3, SSFs = 36%-51%, LOOCV-AUC = 0.691), is substantially improved by the inclusion of DMR17107 and DMR106540 (N = 5, SSFs = 24%-51%, LOOCV-AUC = 0.776). Noteworthy, the LOOCV-AUC can even reach more than 0.8 in expending the model with the two additional but less stable DMR101660 and DMR57278 (N = 7, SSFs = 13%-51%, LOOCV-AUC = 0.806) (Fig. 3). This latter combination provides a sensitivity of 0.818 and a specificity of 0.867. As shown in Table 2 only three of the seven retained DMRs are associated with known genes. These included the Eukaryotic Translation Initiation Factor 2B Subunit 5 (*EIF2B5*), the Ral GTPase Activating Protein Catalytic Alpha Subunit 1 (*RALGAPA1*), but also a noncoding RNA (*LINC01237*), Chromosome 2 Open Reading Frame 81 (*C2orf81*) and Von Willebrand Factor A Domain Containing 5B2 (*VWA5B2*) whose exact functions are still unknown.

**Table 2:** Seven selected DMRs in ERs *vs* NRs with an AUC > 0.85.

DMR ID	FDR	# CpGs	Location	Gene	Mean difference in % methylation	AUC
DMR67206	5.4E−08	45	Intergenic	*NA*	15.99 ± 7.80	0.891
DMR24332	7.2E−12	25	Intron	*LINC01237*	− 9.89 ± 2.77	0.897
DMR30347	1.1E−16	44	TTS	*EIF2B5/ VWA5B2*	16.25 ± 5.44	0.879
DMR17107	1.7E−02	25	Exon	*C2orf81*	12.49 ± 5.16	0.855
DMR106540	3.6E−02	20	Intergenic	*NA*	11.66 ± 3.55	0.842
DMR101660	7.30E−13	15	Promoter-TSS	*RALGAPA1*	− 12.2 ± 3.46	0.842
DMR57278	4.1E−02	33	Intergenic	*NA*	− 3.48 ± 2.55	0.788

For each DMR, the FDR, number of CpGs, location and associated genes are reported. The mean ± standard deviation values were computed using the DNA methylation difference (%) of individual CpG sites between ERs and NRs.

*NA* not available.

Finally, we used the LiPR group to plot the mean methylation levels of the seven DMRs according to ER, NR, and PR status (Supplementary Fig. 2). The visual inspection of box plots identified that the methylation level of LiPRs was always intermediate between the ones of LiERs and LiNRs for the 7 DMRs and all ANOVA between groups were significant (p < 0.016) (detailed results are presented in Supplementary Fig. 2).

## Discussion

To our knowledge, the present study is the first genome-wide analysis of DNA methylation profiles in patients with BD selected for their level of response to long-term treatment with Li. In total blood DNA, we identified 111 significant DMRs in LiERs as compared to LiNRs. The response to Li was mainly associated with methylation changes in genes acting in neuronal compartments. In addition, after applying additional filters (correction for comedications, combinations of univariate and multivariate selections), our study provides an epigenetic signature of response to Li that combines seven DMRs with an AUC above 0.8. If replicated, the excellent discriminative power of this signature suggests that it might be used as a potential biomarker of response to Li.

Within the 111 DMRs identified between LiERs and LiNRs, a significant enrichment in neuronal cell components was found, which was not totally expected from an analysis performed using total blood DNA. Nevertheless, this result is consistent with the literature suggesting a role of Li in neuroprotection and neurogenesis^22,23^; in particular, Li-induced effects on neuroprotection have been associated with hypomethylation at promoter IV of the BDNF gene in rat hippocampal neurons^24^.

The functional significance of the seven DMRs that composed the identified signature are yet to be determined. Only one is located in the promoter/TSS (transcription start site) of *RALGAPA1*, a location usually compatible with a role in gene expression. Three of the other six DMRs are intergenic, one is intronic, one is exonic and the last one is in a TTS (transcription termination site). Of note, DNA methylation has been recently shown to play a role not only in gene repression but also in alternative splicing^25^, transcription elongation^26^ and even in gene transcription activation^27^.

Among the genes associated with the DMRs that composed the signature, none have been directly linked to either BD or response to Li so far. *EIF2B5* is a subunit of eukaryotic translation initiation factor 2B (EIF2B) and therefore a key regulator of protein synthesis^28^. Interestingly, modulation of the brain level of this protein by chronic Li has been reported in rats^29^. Furthermore, a mutant mouse of the *Eif2b5* gene may represent a model for vanishing white matter disease^30^, hence showing a crucial role of this gene in some brain disorders. A long intergenic noncoding RNA is also included in this DNA methylation signature. Long noncoding RNAs are known to regulate gene expression through several mechanisms and it has been suggested that they play significant roles in treatment response in numerous pathologies including psychiatric disorders^31–33^. Of interest, a pharmacogenetic study from the ConLiGen consortium recently identified another noncoding RNA as being associated with Li response^34^. Finally, *RALGAPA1* (also known as *TULIP1*) has been identified as upregulated in the corpus callosum of rats after Li treatment for 4 days^35^. This gene has been proposed as a candidate gene for developmental delay, and may therefore play a role in brain disorders^36^.

One of the strengths of this study is that it provides a genome-wide DNA methylation investigation in LiERs and LiNRs. This is in contrast with the few available studies of the DNA methylation association with response to Li that focused only on one candidate gene, such as *BDNF*^17–19^, or explored global methylation levels without identifying precise locations of DMR^15,16^. The method used in this study has the advantages, unlike microarray results, to have a greater coverage of the genome and to allow to directly count the number of C/T coverage at the CpG sites^37,38^. Another strength is that we chose to restrict this study only to patients with BD type 1, hence with a more homogeneous sample. Indeed, BD subtypes have been found to be associated with the modulation of DNA methylation of specific genes such as *BDNF*^18^. Finally, we attempted to take into account a large number of potential confounding factors that could influence DNA methylation such as gender, age, BMI, smoking status, blood cell composition and found no significant effects from these confounders. More specifically current medications are, by definition, expected to differ between LiERs and LiNRs and may alter DNA methylation levels. In our sample, LiNR participants were more exposed to atypical antipsychotics compared to LiER participants and less exposed to Li. To increase the confidence in our results, we therefore took into account main current psychotropic medications (atypical antipsychotics, antidepressant, anticonvulsants, but also lithium current prescriptions) in the selection of the DMRs of interest.

Nevertheless, several limitations deserve comments. First, in this homogeneous group of patients with BD type I and after the application of selection filters, we were able to identify significant associations, even after correction for multiple testing. However, a larger sample may allow for the detection of additional DMRs of interest and false negative cannot be excluded. Second, this sample has not be pre-selected with specific criteria about the co-medication matrix. Results were adjusted for the possible effect of the number of psychotropic drugs, of each class of medication and current Li treatment. However, the identified set of DMRs might correspond to different treatment regimens. Third, due to the cross-sectional nature of the study, it was not possible to differentiate between Li-induced differences in DNA methylation in LiERs and LiNRs from pre-existing differences due to the genetic background and/or to other environmental factors that might be linked to non-response to Li, such as exposure to childhood maltreatment for example^39^. In addition, the level of disease progression and related characteristics (frequency of mood episodes or suicide attempts) are likely to influence the methylation status^40,41^ and may differ between LiERs and LiNRs participants. Third, this signature is identified in blood, which makes the extrapolation of the biological significance at the brain level uncertain. It has been reported that DNA methylation levels in blood correlates to a limited extent with methylation levels in brain allowing the development of an online database comparing methylation patterns between brain and blood^42^. Among the 207 potential CpGs identified in the selected DMRs only five CpG were present in this database. Blood methylation status of four of these CpGs, in *C2orf81* and *VWA5B2*, were correlated with the methylation status in the brain regions present in the database (Supplementary Fig. 3). In the present state of knowledge it is interesting to note that the 111 identified DMRs in blood DNA are significantly enriched in neuronal components, suggesting that the identified DMRs might give insights into the genes involved in the mechanisms of Li response in neurons.

Although these results were preliminary and would therefore deserve confirmation in independent and larger samples, the identification of a signature of seven DMRs that is able to discriminate between LiERs and LiNRs is promising. Although definitive conclusions regarding the predictive value of this methylomic signature cannot be drawn from this study because of its small sample size and its retrospective design, these results may provide novel candidate DNA regions that should be tested for their methylation status before and after Li initiation in individuals with BD in prospective studies of response to Li. If validated in a prospective study, targeted analysis of DMRs in peripheral blood could be proposed as a biomarker of lithium response in patients with bipolar disorder.

## Methods

### Sample

The samples consisted of French Caucasian individuals who met the DSM-IV criteria for BD type I. Patients were recruited from one academic psychiatric department in France (Paris). Patient inclusion criteria for this study were as follows: aged over 18 years; having a diagnosis of BD type I according to DSM-IV criteria; being Caucasian; and clinically euthymic at the time of inclusion (i.e., having scores for the Montgomery Asberg Depression Rating Scale^43^ and the Young Mania Rating Scale^44^ below five, as well as having had no major mood episodes in the last 3 months). Clinical information was collected using the DIGS (Diagnostic Interview for Genetic Studies)^45^. Written informed consent was obtained from all participants. This study was approved by the French medical ethics committee (Comité de Protection des Personnes (CPP)–IDRCB2008_AO1465_50 VI – Pitié-Salpêtrière 118–08) and carried out according to the approved guidelines. This study is a secondary analysis of the research protocol registered under the number NCT02627404 in ClinicalTrials.gov.

### Response to lithium

For all patients, the response to Li was rated using the ‘Retrospective Criteria of Long-Term Treatment Response in Research Subjects with Bipolar Disorder’, also referred to as the ‘Alda scale’^46^. This scale was specifically developed to allow a retrospective assessment of prophylactic response to treatment in naturalistic conditions. In the present study, in order to maximize the contrast between groups, we chose to compare LiERs versus LiNRs. For this purpose, we used the validated threshold of ≥ 7 on the ALDA total score to define LiERs and ≤ 3 to define LiNRs^8^. 14 partial responders patients with 3 < Alda score < 7 (LiPR for Partial Responders to Li) were not included in the primary analysis (see Supplementary Table 3 for clinical characteristics). The scoring procedure was performed by clinicians trained and approved by the ConLiGen consortium. To reduce the risk of misclassification errors (false positive and false negative cases), all patients were selected because they all received Li for at least two consecutive years and exhibit good compliance (score of 0 or 1 at the item B4). Groups of LiERs and LiNRs have been matched on age, gender, BMI (Body Mass Index) and tobacco use in order to minimize the influence of these potential confounding factors in DNA methylation studies^47^.

### SeqCap Epi methylation

MethylCap-seq was performed using the SeqCap Epi CpGiant Enrichment kit (Roche Diagnostics, France) according to the manufacturer’s directions to target 80.5 Mb of the human genome with > 5.5 million CpGs^48^. Briefly, genomic DNA (1 μg) was randomly sheared to 200 bp (average fragment size) using a Bioruptor Pico (Diagenode, France). Bisulfite conversion was performed using EZ DNA Methylation-Lightning Kit (Zymo Research, France). Libraries were generated using KAPA Lib Prep Kit (Illumina,Roche). PCR enrichement was performed using the SeqCap Epi oligo pool as directed in the protocol. After purification, quality and quantification were performed using a Labchip GX (Perkin Elmer, France) and Qubit.

### Data processing and methylation analysis

Quality control of the raw sequencing data was conducted with the FastQC program v0.11.4. Adapter sequences were removed and poor quality reads were filtered using Trimmomatic v0.35. Trimmed reads were then aligned using the software suite Bismark v0.16.1. The Human hg19 reference genome was downloaded from the UCSC FTP server (ftp://hgdownload.soe.ucsc.edu/goldenPath/hg19/chromosomes/).

All cytosines on the reference genome were converted to thymines for both strands using the bismark_genome_preparation command, and the reference index was built using Bowtie2 v2.2.6. All Paired-end reads were then mapped to this modified genome using the Bismark option—bowtie2 with standard parameters. The resulting BAM files were processed by deduplicate_bismark to remove PCR duplication artifacts; methylation information by sites was then extracted with bismark_methylation_extractor. As a filtering step to exclude the low-coverage CpGs, only CpG sites with a minimal coverage of 5 × in at least 80% of samples per group were considered for the subsequent analysis. The number of CpG captured and average of read per sample is presented in Supplementary Table 4.

Detection of DMRs was performed using the metilene software v0.2–6^49^. First, the output of Bismark was converted to metilene input format (CpG methylation ratios). DMRs identification was obtained using the following criteria: a DMR should comprise at least 5 consecutive CpG sites, with a sequencing depth > 5 and a maximum distance between CpGs of < 100 bp. Significant DMRs were identified using the Mann–Whitney-U test at a false discovery rate (FDR) < 0.05.

### Blood cell-type composition

Blood cell-type composition analysis was performed using R software (version 3.5.2, https://cran.r-project.org/) to apply the deconvolution method by Houseman and colleagues^50^ with the reference Reinius dataset^51^. The overall proportions of CD4 + T cells, CD8 + T cells, B cells, natural killer cells, monocytes and granulocytes were then determined using the function projectCellType() in the minfi R Bioconductor package^52^ with the filtered CpG sites that overlap with 600 cell-type-specific CpGs previously selected by Jaffe and Irrizary^53^ on the Illumina 450 k platform. Based on the estimated proportions, differences in blood cell-type composition between the groups were tested for each cell type by t-test at a significance level of < 0.05.

### Pathway enrichment

Gene ontology (GO) functional annotation of the genes at the top of the DMR list was performed using the DAVID 6.8 web tool^54,55^ to obtain the most enriched GO terms of the cellular component category (GOTERM_CC_ALL) at a significance level of < 0.05.

### Statistical analysis

All statistical analyses were performed using R version 3.5.2 {https://www.R-project.org}. The Mann–Whitney-U, chi-square and Fisher’s exact tests were used to examine differences between the two groups using a significance level of < 0.05.

Following the methylation analysis described above, additional filters were applied to select DMRs and to control for the influence of comedications among participants (atypical antipsychotics, antidepressants or anticonvulsants). Since by definition, LiNRs are less likely to receive Li, but other mood stabilizers, a further filter was applied based on the current prescription of Li. To do so, binary logistic regressions were performed to obtain a shorter list of DMRs with a p-value < 0.1 in the univariate analysis. To account for potential confounding comedication effect, all the models were compared with and without each comedication factor. Thus, all DMRs with an adjusted odds ratio (OR) falling outside the 95% confidence limits of the crude (unadjusted) OR were excluded to avoid a nonspecific effect of a medication.

Finally, the ability to distinguish between LiER and LiNR with the preselected DMRs was studied through a multivariate analysis. A model was developed using sparse partial least squares discriminant analysis (sPLS-DA) as implemented in the splsda function of the R package mixOmics^56^. sPLS-DA is a component-based method that combines Partial Least Squares regression to discriminate between the Li groups, and Lasso penalization to select a subset of the most relevant DMRs. Using the R package pROC, the optimal number of DMRs to include in the first component was determined through a receiver operating characteristic (ROC) curve analysis with the area under the curve (AUC) statistics. Based on the Lasso method, the list of the selected DMRs was thus expanded one-by-one until the smallest combination of DMRs maximizing the AUC criterion was obtained. To further assess the stability of the selected DMRs, the feature selection with sPLS-DA was repeated on 1,000 bootstrap samples of the same size as the original dataset. Because the number of patients was too limited with no independent test set, the ROC curves and AUCs were derived using leave-one-out cross-validation (LOOCV) to avoid over-optimistic performance estimates.

Based on methylation data from all patients (LiER, LiNR and LiPR), boxplots were generated to examine the methylation status of the LiPRs as compared to the two other groups and tested with ANOVA.

## Supplementary information

Supplementary file1 (PDF 1015 kb)
