# Supplementary material for: A DNA methylation signature discriminates between excellent and non-response to lithium in patients with bipolar disorder type 1
Source: Sci Rep. 2020 Jul 22;10:12239. doi: 10.1038/s41598-020-69073-0 (PMC7376060; doi:10.1038/s41598-020-69073-0)
Supplement: Supplementary file 1 — Supplementary file1 (PDF 1015 kb) [file 41598_2020_69073_MOESM1_ESM.pdf]

A DNA methylation signature discriminates between excellent and non-response to lithium in patients with bipolar disorder type 1

Running title: DNA methylation and response to lithium in BD

Marie-Claire C [1], Lejeune FX [5], Mundwiller E [4], Ulveling D [5], Moszer I [5], Bellivier F *[1,2,3,6], Etain B[1,2,3,6]

1) Optimisation thérapeutique en neurospsychopharmacologie, INSERM U1144, Université de Paris, Université Sorbonne Paris Cité, Paris, France ;

2) AP-HP, GH Saint-Louis – Lariboisière – F. Widal, Pole de Psychiatrie et de Médecine Addictologique, Paris, France;

3) Fondation FondaMental, Créteil, France;

4) IGenSeq, Institut du Cerveau et de la Moelle Épinière, Paris, France;

5) Bioinformatics and Biostatistics Core Facility iCONICS, Inserm U 1127, CNRS UMR 7225, Sorbonne Université UMR S 1127, Institut du Cerveau et de la Moelle Épinière, Paris, France ;

6) Université de Paris, faculté de Médecine, Paris, France.

* Corresponding author: cynthia.marie-claire@parisdescartes.fr

Supplementary Figure 1: Predicted proportions of T lymphocytes (CD8T and CD4T), Natural killer cells (NK), B Lymphocytes (Bcell), Monocytes (Mono) and granulocytes (Gran) in periphal blood samples from Li excellent responders and non responders.

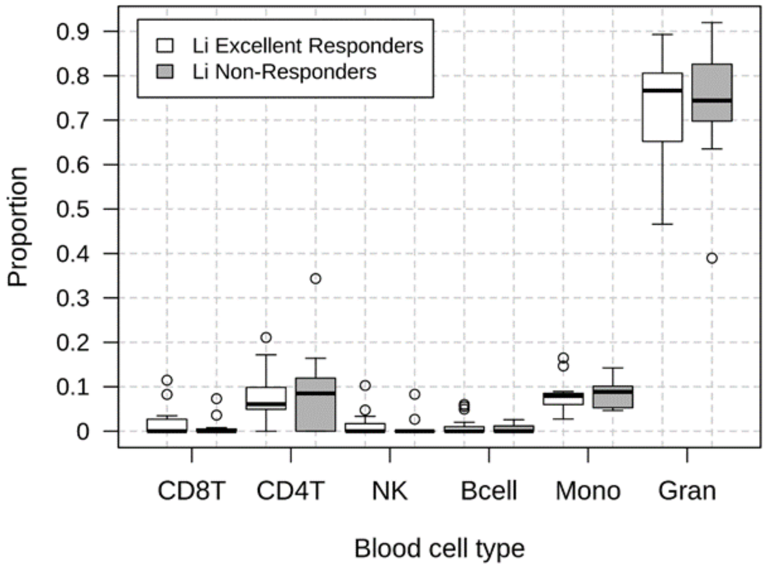

Supplementary Figure 2: Box plots of the individual methylation levels of the 7 DMRs in the 15 LiER and 11 LiNR included in the initial analysis and additional 14 LiPR. Results of the one way ANOVA (Welch's) are presented for each DMR.

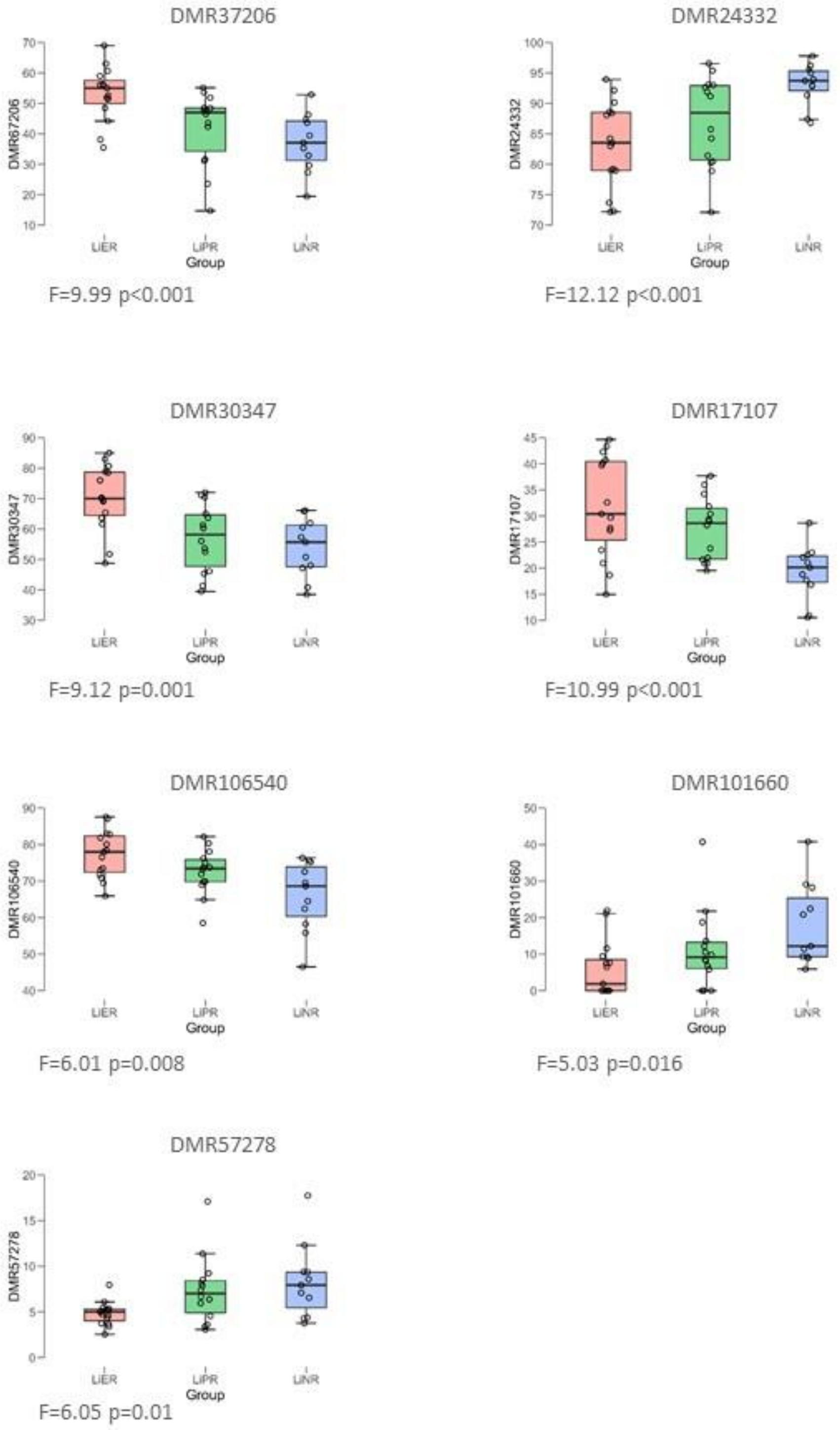

Supplementary Figure 3: Correlation (Spearman correlation value) of blood and three brain regions methylation for the five CpG present in the Blood-Brain Epigenetic Concordance (BECon) database. Values were generated from the Blood-Brain Epigenetic Concordance database (https://redgar598.shinyapps.io /BECon/). BA10 (Broadmann area 10, prefrontal cortex; BA7 (Broadmann area 7, parietal cortex), BA20 (Broadmann area 20, temporal cortex).

	Chr	Coor	Gene(s)	Gene Region(s)	Variability				Correlation			Cell Composition	
					BA10	BA20	BA7	Blood	BA10	BA20	BA7	Blood	Brain
cg03292743	2	74642838	C2orf81	intragenic	0.1	0.08	0.1	0.22	0.44	0.04	0.32	0.03	0.01
cg04490945	3	183959465	MIR1224, VWA5B2, MIR1224	ree_plus, intragenic, promot	0.07	0.07	0.04	0.15	-0.22	0.17	0.19	0.03	0.01
cg16625004	3	183959550	MIR1224, VWA5B2	three_plus, intragenic	0.04	0.06	0.04	0.08	0.4	0.26	0.01	0.02	0
cg08411860	3	183959746	VWA5B2	intragenic	0.07	0.07	0.05	0.13	0.08	0.27	-0.41	0.03	0.01
cg24363374	3	183959853	VWA5B2, ALG3	three_plus, three_plus	0.09	0.1	0.1	0.15	0.29	0.44	0.04	0.04	0.02

Correlation or Cell Composition Percentile or Variability Status

- 90% (Positive)
- 75-90% (Positive)
- 50-75% (Positive)
- <50% (Positive)
- <50% (Negative)
- 50-75% (Negative)
- 75-90% (Negative)
- 90% (Negative)
- 90% (Blood Cell Comp.)
- 75-90% (Blood Cell Comp.)
- 50-75% (Blood Cell Comp.)
- <50% (Blood Cell Comp.)
- 90% (Brain Cell Comp.)
- 75-90% (Brain Cell Comp.)
- 50-75% (Brain Cell Comp.)
- <50% (Brain Cell Comp.)
- Not Variable
- Variable
- Genomic Info

Supplementary Table 1: List of the cell type specific CpGs used for blood cell types determination

List of 600 cell type specific CpGs (Jaffe & Irizarry, Genome Biol. 2014)	CpGs used in BD methylation analysis
cg00058449	TRUE
cg00091349	TRUE
cg00103299	TRUE
cg00149213	TRUE
cg00159243	TRUE
cg00208012	TRUE
cg00219921	FALSE
cg00221794	TRUE
cg00287915	TRUE
cg00288598	TRUE
cg00366435	TRUE
cg00369058	TRUE
cg00439981	TRUE
cg00440980	TRUE
cg00445959	FALSE
cg00458681	TRUE
cg00619207	TRUE
cg00629585	TRUE
cg00644351	TRUE
cg00660167	TRUE
cg00661777	TRUE
cg00701951	TRUE
cg00702872	TRUE
cg00705730	TRUE
cg00719568	TRUE
cg00760938	TRUE
cg00782811	TRUE
cg00795812	TRUE
cg00813999	TRUE
cg00970435	TRUE
cg00984060	FALSE
cg01002223	TRUE
cg01024458	TRUE
cg01040649	TRUE
cg01154505	FALSE
cg01156249	TRUE
cg01367627	TRUE

cg01383401	TRUE
cg01447281	FALSE
cg01525879	TRUE
cg01582066	TRUE
cg01692842	TRUE
cg01710351	TRUE
cg01874152	TRUE
cg01881899	TRUE
cg01940139	TRUE
cg01987702	TRUE
cg02016178	TRUE
cg02021288	FALSE
cg02150910	TRUE
cg02212339	TRUE
cg02212836	TRUE
cg02237342	TRUE
cg02256631	FALSE
cg02321062	TRUE
cg02331830	TRUE
cg02366988	TRUE
cg02384859	TRUE
cg02402946	FALSE
cg02573468	TRUE
cg02598071	TRUE
cg02606840	TRUE
cg02661764	TRUE
cg02665297	TRUE
cg02720566	TRUE
cg02734358	TRUE
cg02821342	TRUE
cg02861441	TRUE
cg02861504	TRUE
cg02964396	FALSE
cg03055671	FALSE
cg03077671	TRUE
cg03146219	TRUE
cg03196485	TRUE
cg03209642	FALSE
cg03268893	TRUE
cg03278514	TRUE
cg03313271	TRUE
cg03318654	TRUE
cg03340036	TRUE
cg03347559	TRUE
cg03379131	TRUE
cg03419136	TRUE

cg03429643	TRUE
cg03538296	TRUE
cg03605454	TRUE
cg03637218	TRUE
cg03708221	TRUE
cg03722295	TRUE
cg03795574	TRUE
cg03861966	TRUE
cg03887528	TRUE
cg03889044	TRUE
cg03984502	TRUE
cg03989617	FALSE
cg03992638	TRUE
cg04050463	FALSE
cg04153882	TRUE
cg04244970	TRUE
cg04276058	TRUE
cg04307083	TRUE
cg04329870	TRUE
cg04453471	TRUE
cg04468741	TRUE
cg04606170	TRUE
cg04609694	TRUE
cg04658357	TRUE
cg04828493	FALSE
cg04838847	TRUE
cg04855678	FALSE
cg04858164	FALSE
cg04859102	TRUE
cg04910877	TRUE
cg04947157	TRUE
cg04978343	TRUE
cg05041073	TRUE
cg05070273	TRUE
cg05205074	TRUE
cg05298628	TRUE
cg05371506	TRUE
cg05398700	FALSE
cg05492387	FALSE
cg05496363	TRUE
cg05517541	TRUE
cg05579731	TRUE
cg05604112	TRUE
cg05617307	TRUE
cg05627557	FALSE
cg05653887	TRUE

cg05723552	TRUE
cg05859076	TRUE
cg05870586	TRUE
cg05875239	TRUE
cg05923857	TRUE
cg05936004	FALSE
cg06015525	TRUE
cg06040872	TRUE
cg06117093	TRUE
cg06202778	TRUE
cg06270401	FALSE
cg06317348	FALSE
cg06329392	FALSE
cg06398474	TRUE
cg06419846	TRUE
cg06449334	TRUE
cg06465076	TRUE
cg06470558	TRUE
cg06489037	TRUE
cg06526020	TRUE
cg06690168	TRUE
cg06889975	TRUE
cg06975311	TRUE
cg07002540	TRUE
cg07016730	TRUE
cg07019638	TRUE
cg07196673	TRUE
cg07212327	TRUE
cg07248223	TRUE
cg07307830	TRUE
cg07363543	TRUE
cg07427475	FALSE
cg07489413	TRUE
cg07546943	TRUE
cg07584558	TRUE
cg07590102	TRUE
cg07597976	TRUE
cg07616394	TRUE
cg07635227	FALSE
cg07661835	TRUE
cg07675477	TRUE
cg07679948	TRUE
cg07698804	TRUE
cg07700837	TRUE
cg07702659	TRUE
cg07721872	TRUE

cg07725527	FALSE
cg07728865	TRUE
cg07746532	TRUE
cg07754940	TRUE
cg07809589	FALSE
cg07837085	TRUE
cg07881210	TRUE
cg07930752	FALSE
cg07970799	TRUE
cg07978738	TRUE
cg08130572	FALSE
cg08154963	FALSE
cg08224785	FALSE
cg08253808	TRUE
cg08280936	TRUE
cg08506127	TRUE
cg08549335	TRUE
cg08560387	FALSE
cg08608952	TRUE
cg08635931	TRUE
cg08645860	FALSE
cg08661469	TRUE
cg08682625	TRUE
cg08694574	TRUE
cg08698943	TRUE
cg08769189	TRUE
cg08777095	FALSE
cg08817540	TRUE
cg08864105	TRUE
cg08884752	TRUE
cg08908131	TRUE
cg09220326	TRUE
cg09221159	TRUE
cg09257735	FALSE
cg09261289	TRUE
cg09300089	TRUE
cg09318840	TRUE
cg09322573	TRUE
cg09342997	TRUE
cg09421111	FALSE
cg09490277	TRUE
cg09543427	TRUE
cg09610286	TRUE
cg09654116	FALSE
cg09662852	TRUE
cg09712234	TRUE

cg09849688	FALSE
cg09859659	TRUE
cg09958801	FALSE
cg09960724	TRUE
cg09993145	FALSE
cg10054641	TRUE
cg10057264	TRUE
cg10096215	TRUE
cg10142452	TRUE
cg10274029	TRUE
cg10278149	TRUE
cg10373891	TRUE
cg10480329	FALSE
cg10499974	TRUE
cg10502324	TRUE
cg10505873	FALSE
cg10516886	TRUE
cg10536999	TRUE
cg10578938	TRUE
cg10611016	TRUE
cg10624395	TRUE
cg10628126	TRUE
cg10639435	TRUE
cg10699171	TRUE
cg10761804	TRUE
cg10768932	TRUE
cg10782923	TRUE
cg10812236	TRUE
cg10825315	TRUE
cg10825530	TRUE
cg10837404	FALSE
cg10837858	TRUE
cg10879992	TRUE
cg10880902	TRUE
cg10944833	FALSE
cg11014468	TRUE
cg11067179	TRUE
cg11070172	TRUE
cg11163777	TRUE
cg11176694	TRUE
cg11186858	TRUE
cg11230435	TRUE
cg11354682	TRUE
cg11411904	TRUE
cg11465943	TRUE
cg11525052	TRUE

**cg11531557**	FALSE
**cg11557901**	TRUE
**cg11637084**	TRUE
**cg11661493**	TRUE
**cg11671771**	FALSE
**cg11774624**	TRUE
**cg11811510**	TRUE
**cg11837293**	FALSE
**cg11906021**	FALSE
**cg11944101**	TRUE
**cg11977716**	FALSE
**cg11978441**	TRUE
**cg11992736**	FALSE
**cg12032027**	TRUE
**cg12105450**	TRUE
**cg12177944**	TRUE
**cg12182124**	TRUE
**cg12207930**	TRUE
**cg12294633**	TRUE
**cg12297231**	FALSE
**cg12344862**	FALSE
**cg12403778**	TRUE
**cg12477370**	TRUE
**cg12480903**	TRUE
**cg12483340**	TRUE
**cg12531611**	TRUE
**cg12655112**	TRUE
**cg12793123**	TRUE
**cg12838644**	FALSE
**cg12996622**	TRUE
**cg13027206**	FALSE
**cg13066703**	TRUE
**cg13068915**	FALSE
**cg13093111**	TRUE
**cg13133835**	FALSE
**cg13203135**	TRUE
**cg13209388**	TRUE
**cg13315450**	FALSE
**cg13356455**	TRUE
**cg13430807**	FALSE
**cg13437525**	TRUE
**cg13449535**	TRUE
**cg13468144**	TRUE
**cg13525276**	TRUE
**cg13543915**	FALSE
**cg13595556**	FALSE

**cg13617280**	TRUE
**cg13651908**	TRUE
**cg13652008**	TRUE
**cg13695076**	TRUE
**cg13738327**	TRUE
**cg13785123**	TRUE
**cg13828440**	TRUE
**cg13924996**	FALSE
**cg13947995**	TRUE
**cg14011327**	TRUE
**cg14032529**	TRUE
**cg14082886**	FALSE
**cg14084689**	FALSE
**cg14109551**	TRUE
**cg14135025**	FALSE
**cg14145194**	TRUE
**cg14284211**	TRUE
**cg14301562**	TRUE
**cg14317609**	FALSE
**cg14331899**	TRUE
**cg14364797**	FALSE
**cg14384416**	TRUE
**cg14477767**	TRUE
**cg14482811**	TRUE
**cg14496375**	FALSE
**cg14555759**	FALSE
**cg14564351**	TRUE
**cg14574996**	FALSE
**cg14583973**	TRUE
**cg14595786**	TRUE
**cg14655843**	TRUE
**cg14729344**	FALSE
**cg14936008**	TRUE
**cg15014975**	TRUE
**cg15029631**	TRUE
**cg15035590**	TRUE
**cg15086439**	TRUE
**cg15087459**	TRUE
**cg15114607**	TRUE
**cg15153141**	TRUE
**cg15241779**	TRUE
**cg15258980**	TRUE
**cg15348679**	TRUE
**cg15384400**	TRUE
**cg15459165**	TRUE
**cg15474337**	TRUE

**cg15532942**	FALSE
**cg15535471**	TRUE
**cg15555970**	FALSE
**cg15564619**	TRUE
**cg15595502**	TRUE
**cg15657641**	TRUE
**cg15691199**	TRUE
**cg15791438**	TRUE
**cg15821562**	TRUE
**cg15878616**	TRUE
**cg15906007**	TRUE
**cg15956469**	FALSE
**cg16013543**	TRUE
**cg16047279**	FALSE
**cg16148346**	TRUE
**cg16210395**	TRUE
**cg16212145**	TRUE
**cg16235995**	FALSE
**cg16256390**	FALSE
**cg16429499**	TRUE
**cg16519762**	FALSE
**cg16582517**	TRUE
**cg16596052**	FALSE
**cg16636767**	FALSE
**cg16654610**	TRUE
**cg16681873**	TRUE
**cg16802439**	TRUE
**cg16836311**	TRUE
**cg16893868**	FALSE
**cg16941568**	FALSE
**cg17117981**	TRUE
**cg17202724**	TRUE
**cg17232476**	TRUE
**cg17266282**	FALSE
**cg17343167**	FALSE
**cg17346246**	TRUE
**cg17638468**	TRUE
**cg17694130**	FALSE
**cg17768768**	TRUE
**cg17830204**	FALSE
**cg17851021**	TRUE
**cg17852482**	TRUE
**cg17879722**	TRUE
**cg17995557**	TRUE
**cg18050634**	FALSE
**cg18055007**	TRUE

cg18066690	TRUE
cg18067520	TRUE
cg18081760	FALSE
cg18128887	FALSE
cg18149207	TRUE
cg18154117	TRUE
cg18174654	FALSE
cg18184053	TRUE
cg18224038	TRUE
cg18250453	FALSE
cg18310515	TRUE
cg18437209	FALSE
cg18546384	TRUE
cg18582260	TRUE
cg18672716	TRUE
cg18701685	FALSE
cg18734095	FALSE
cg18800235	TRUE
cg18830527	TRUE
cg18835472	TRUE
cg18857618	TRUE
cg18865207	TRUE
cg18881723	TRUE
cg18886071	TRUE
cg18923906	FALSE
cg18990407	TRUE
cg19037107	TRUE
cg19055828	TRUE
cg19066520	TRUE
cg19269039	TRUE
cg19276014	TRUE
cg19296305	TRUE
cg19301114	TRUE
cg19358608	TRUE
cg19387310	TRUE
cg19408207	TRUE
cg19410791	TRUE
cg19455189	TRUE
cg19460836	TRUE
cg19594892	FALSE
cg19612309	TRUE
cg19679865	TRUE
cg19696012	TRUE
cg19730972	TRUE
cg19835973	TRUE
cg19927257	FALSE

cg19933595	TRUE
cg20146241	FALSE
cg20450123	TRUE
cg20460771	TRUE
cg20525486	TRUE
cg20602300	FALSE
cg20610950	TRUE
cg20641531	TRUE
cg20657709	TRUE
cg20660269	TRUE
cg20681931	FALSE
cg20737812	TRUE
cg20786131	TRUE
cg20978247	TRUE
cg21057323	TRUE
cg21091766	TRUE
cg21220520	TRUE
cg21221767	TRUE
cg21248060	FALSE
cg21254939	TRUE
cg21268578	FALSE
cg21285555	TRUE
cg21291385	TRUE
cg21333217	TRUE
cg21350349	TRUE
cg21467614	TRUE
cg21474838	TRUE
cg21565496	TRUE
cg21596498	TRUE
cg21634628	TRUE
cg21671607	TRUE
cg21683390	TRUE
cg21685770	FALSE
cg21743182	TRUE
cg21761853	TRUE
cg21792134	TRUE
cg22001211	TRUE
cg22014398	FALSE
cg22065498	FALSE
cg22153728	TRUE
cg22212560	FALSE
cg22222999	TRUE
cg22381196	FALSE
cg22498365	TRUE
cg22563987	TRUE
cg22620363	TRUE

**cg22632840**	FALSE
**cg22651103**	TRUE
**cg22708902**	FALSE
**cg22749642**	TRUE
**cg22903735**	TRUE
**cg22907103**	FALSE
**cg23045908**	FALSE
**cg23053742**	TRUE
**cg23169883**	TRUE
**cg23213217**	TRUE
**cg23244761**	TRUE
**cg23272399**	TRUE
**cg23325335**	TRUE
**cg23357533**	TRUE
**cg23357708**	TRUE
**cg23358740**	TRUE
**cg23391288**	TRUE
**cg23398076**	TRUE
**cg23624705**	TRUE
**cg23643435**	TRUE
**cg23661721**	TRUE
**cg23673974**	TRUE
**cg23683962**	TRUE
**cg23723835**	TRUE
**cg23763137**	TRUE
**cg23792308**	TRUE
**cg23819411**	TRUE
**cg23954655**	TRUE
**cg24088496**	TRUE
**cg24238409**	TRUE
**cg24345747**	TRUE
**cg24365417**	TRUE
**cg24430034**	TRUE
**cg24462702**	TRUE
**cg24641737**	TRUE
**cg24662823**	TRUE
**cg24788483**	TRUE
**cg24838825**	TRUE
**cg24904788**	TRUE
**cg24957609**	TRUE
**cg25006077**	TRUE
**cg25104621**	TRUE
**cg25131632**	TRUE
**cg25235205**	FALSE
**cg25350872**	FALSE
**cg25383568**	TRUE

cg25416125	TRUE
cg25486399	TRUE
cg25496930	TRUE
cg25518868	TRUE
cg25535316	FALSE
cg25554283	TRUE
cg25598890	TRUE
cg25599673	TRUE
cg25600606	TRUE
cg25605731	TRUE
cg25616055	TRUE
cg25683989	TRUE
cg25719851	TRUE
cg25729350	TRUE
cg25739938	TRUE
cg25848037	TRUE
cg25856090	TRUE
cg25887844	TRUE
cg25893560	TRUE
cg25898577	TRUE
cg25939861	TRUE
cg26012482	TRUE
cg26056277	TRUE
cg26121053	TRUE
cg26166854	TRUE
cg26169783	FALSE
cg26227465	TRUE
cg26270899	TRUE
cg26341831	TRUE
cg26538782	FALSE
cg26552743	TRUE
cg26562691	TRUE
cg26572165	TRUE
cg26692003	TRUE
cg26707718	FALSE
cg26795340	TRUE
cg26872907	TRUE
cg26896499	FALSE
cg26971042	TRUE
cg26986871	TRUE
cg27027151	TRUE
cg27043010	FALSE
cg27058497	TRUE
cg27059140	TRUE
cg27106643	TRUE
cg27118278	FALSE

**cg27122888**	TRUE
**cg27166718**	TRUE
**cg27202779**	FALSE
**cg27309871**	TRUE
**cg27366072**	TRUE
**cg27423838**	TRUE
**cg27485845**	TRUE
**cg27506442**	TRUE
**cg27507284**	TRUE
**cg27582527**	TRUE
**ch.17.480931F**	FALSE

Supplementary Table 2: List of the 74 genes associated with the 111 identified DMRs (FDR 0.05)

**Official gene symbol**	**Name**	**Chromosome**
ACAT2	acetyl-CoA acetyltransferase 2(ACAT2)	6
ACKR3	atypical chemokine receptor 3(ACKR3)	2
ADGB	androglobin(ADGB)	6
ANKRD20A7P	ankyrin repeat domain 20 family member A7, pseudogene(ANKRD20A7P)	9
ARMC10	armadillo repeat containing 10(ARMC10)	7
ARRB2	arrestin beta 2(ARRB2)	17
ARSE	arylsulfatase E (chondrodysplasia punctata 1)(ARSE)	X
BLACE	B-cell acute lymphoblastic leukemia expressed(BLACE)	7
C2orf81	chromosome 2 open reading frame 81(C2orf81)	2
C5orf17	chromosome 5 open reading frame 17(C5orf17)	5
C6orf25	chromosome 6 open reading frame 25(C6orf25)	6
CCDC144NL	coiled-coil domain containing 144 family, N-terminal like(CCDC144NL)	17
CFDP1	craniofacial development protein 1(CFDP1)	16
CRYGEP	crystallin gamma E, pseudogene(CRYGEP)	2
CUZD1	CUB and zona pellucida like domains 1(CUZD1)	10
DLGAP2||ERICH1-AS1	DLG associated protein 2(DLGAP2)	8
DPYS	dihydropyrimidinase(DPYS)	8
DUSP22	dual specificity phosphatase 22(DUSP22)	6
EBF3	early B-cell factor 3(EBF3)	10
EEPD1	endonuclease/exonuclease/phosphatase family domain containing 1(EEPD1)	7
EIF1AXP1	eukaryotic translation initiation factor 1A, X-linked pseudogene 1(EIF1AXP1)	1
EIF2B5	eukaryotic translation initiation factor 2B subunit epsilon(EIF2B5)	3
EPHA6	EPH receptor A6(EPHA6)	3
ESPNP	espin pseudogene(ESPNP)	1
FAAP20||C1orf86	Fanconi anemia core complex associated protein 20(FAAP20)	1
FAM24B	family with sequence similarity 24 member B(FAM24B)	10
FBXL13	F-box and leucine rich repeat protein 13(FBXL13)	7
FIP1L1	factor interacting with PAPOLA and CPSF1(FIP1L1)	4
FN3K	fructosamine 3 kinase(FN3K)	17
GFPT2	glutamine-fructose-6-phosphate transaminase 2(GFPT2)	5
HLA-DRB9	major histocompatibility complex, class II, DR beta 9 (pseudogene)(HLA-DRB9)	6
HOOK2	hook microtubule tethering protein 2(HOOK2)	19
HOXA3	homeobox A3(HOXA3)	7
HOXA4	homeobox A4(HOXA4)	7
HOXA-AS2	HOXA cluster antisense RNA 2(HOXA-AS2)	7
HOXA-AS3	HOXA cluster antisense RNA 3(HOXA-AS3)	7
HOXB3	homeobox B3(HOXB3)	17
HOXB6	homeobox B6(HOXB6)	17
HOXB-AS3	HOXB cluster antisense RNA 3(HOXB-AS3)	17
IGF2BP1	insulin like growth factor 2 mRNA binding protein 1(IGF2BP1)	17
KATNBL1P6	katanin regulatory subunit B1 like 1 pseudogene 6(KATNBL1P6)	6
LINC01237	long intergenic non-protein coding RNA 1237(LINC01237)	2
LRCOL1	leucine rich colipase like 1(LRCOL1)	12
LZTS3	leucine zipper tumor suppressor family member 3(LZTS3)	20

MEI1	meiotic double-stranded break formation protein 1(MEI1)	22
MYT1L	myelin transcription factor 1 like(MYT1L)	2
NBPF13P	neuroblastoma breakpoint family member 13, pseudogene(NBPF13P)	1
NLRP1	NLR family pyrin domain containing 1(NLRP1)	17
OR2L13	olfactory receptor family 2 subfamily L member 13(OR2L13)	1
P4HA2	prolyl 4-hydroxylase subunit alpha 2(P4HA2)	5
PACRG	PARK2 coregulated(PACRG)	6
PDLIM4	PDZ and LIM domain 4(PDLIM4)	5
PKP3	plakophilin 3(PKP3)	11
PM20D1	peptidase M20 domain containing 1(PM20D1)	1
POU6F2	POU class 6 homeobox 2(POU6F2)	7
PRDM16	PR/SET domain 16(PRDM16)	1
PRRT3	proline rich transmembrane protein 3(PRRT3)	3
PRRT3-AS1	PRRT3 antisense RNA 1(PRRT3-AS1)	3
PTPRN2	protein tyrosine phosphatase, receptor type N2(PTPRN2)	7
RAD51B	RAD51 paralog B(RAD51B)	14
RALGAPA1	Ral GTPase activating protein catalytic alpha subunit 1(RALGAPA1)	14
SCRIB	scribbled planar cell polarity protein(SCRIB)	8
SOD2	superoxide dismutase 2, mitochondrial(SOD2)	6
STK32C	serine/threonine kinase 32C(STK32C)	10
TCP10L2	t-complex 10-like 2(TCP10L2)	6
TENM2	teneurin transmembrane protein 2(TENM2)	5
TMEM131	transmembrane protein 131(TMEM131)	2
TPGS1	tubulin polyglutamylase complex subunit 1(TPGS1)	19
VIPR2	vasoactive intestinal peptide receptor 2(VIPR2)	7
VWA5B2	von Willebrand factor A domain containing 5B2(VWA5B2)	3
WSCD1	WSC domain containing 1(WSCD1)	17
ZNF331	zinc finger protein 331(ZNF331)	19
ZNF714	zinc finger protein 714(ZNF714)	19
ZPBP	zona pellucida binding protein(ZPBP)	7

Supplementary Table 3: Clinical characteristics of all the patients with bipolar disorder included in the global methylation study

	**LiER**	**LiPR**	**LiNR**
N	15	14	11
Alda range	7-10	4-6	0-3
Ratio Male/Female	8/7	4/10	6/5
Age	49.85 +/- 11.24	46.67 +/- 9.63	46.58 +/- 6.54
BD type 1	100%	100%	100%
BMI	24.48 +/- 3.26	25.88 +/- 5.43	25.71 +/- 3.16
Smokers yes/no	6/9	8/6	6/5
Current medication			
Li yes/no	14/1	12/0	5/6
Anticonvulsants yes/no	3/12	6/8	5/6
Atypical antipsychotic yes/no	0/15	6/8	7/4
Antidepressants yes/no	3/12	4/10	4/7
Number of psychotropic drugs 1/2/3	11/3/1	0/11/3	3/4/4

Supplementary Table 4: Number of CpGs captured and average read depth of each of the CpGs per sample.

sample	nb_CpGs	mean_coverage
GAN-001-620	3686657	17.89
GAN-001-621	3684864	15.81
GAN-001-628	3686607	18.34
GAN-007-013	3686703	17.53
GAN-007-018	3685961	15.37
GAN-007-020	3686832	18.19
GAN-007-022	3679576	14.65
GAN-007-040	3685557	16.65
GAN-007-047	3686785	18.71
GAN-007-048	3686755	18.26
GAN-007-055	3685091	20.62
GAN-007-057	3686682	19.26
BP-440	3686649	18.2
BP-516	3678799	11.62
BB-204	3686560	17.94
BB-205	3686367	17.11
GAN-001-627	3686135	17.57
GAN-007-017	3686940	18.67
GAN-007-024	3682032	16.37
GAN-007-027	3686195	17.15
GAN-007-035	3686793	18.6
GAN-007-036	3686476	17.04
GAN-007-043	3683749	18.65
GAN-007-044	3674526	9.7
GAN-007-054	3686457	17.75
GAN-007-058	3684267	14.15
GAN-007-062	3687031	20.18
BP-231	3686061	17.54
